# Nanomaterials in Cancer Diagnosis and Therapy

**DOI:** 10.3390/ijms232213770

**Published:** 2022-11-09

**Authors:** Francesca Brero, Salvatore Gallo

**Affiliations:** 1 Istituto Nazionale di Fisica Nucleare (INFN), Sezione di Pavia, Via Agostino Bassi, 6, 27100 Pavia, Italy; 2 National Interuniversity Consortium of Materials Science and Technology (INSTM), Via G. Giusti, 9, 50121 Firenze, Italy; 3 Dipartimento di Fisica “Aldo Pontremoli”, Università di Milano, Via Giovanni Celoria, 16, 20133 Milano, Italy; 4 Istituto Nazionale di Fisica Nucleare (INFN), Sezione di Milano, Via Giovanni Celoria, 16, 20133 Milano, Italy

## 1. Introduction

Currently, the most commonly used treatments for cancer are surgery, radiotherapy, and chemotherapy [1,2,3,4,5,6]. All three methods could lead to damaging normal tissues or the incomplete eradication of tumors. In light of this, significant interest has arisen towards developing innovative strategies that are better tailored to treating this pathology. Nanotechnologies offer the means to (i) guide the surgical resection of tumors; (ii) target chemotherapies directly and selectively to cancerous cells; and (iii) enhance the therapeutic efficacy of radiation-based and other current treatment modalities (e.g., combined techniques). All of this will benefit patients, who will suffer fewer side effects and have higher survival rates with a better quality of life.

Furthermore, nanomaterials offer a wide gamut of interesting properties, such as the possibility of getting close to a biological entity of interest or that of being easily brought inside a patient’s body. For nanomaterials to be applied, a combination of different scientific branches is often required, among them being biology, chemistry, physics, medicine, and engineering. The goal is to develop new tools that might increase the quality of diagnostic images and increase the effectiveness of anticancer treatments. This current Special Issue is a thorough collection of articles dealing with the synthesis and characterization of nanomaterials, of which the authors show the mechanisms of action and prospective applications for therapies and/or diagnoses, both in vitro and in vivo. For this purpose, the content includes basic, translational, and clinical research addressing the diagnosis, treatment, monitoring, prediction, and prevention of diseases. Furthermore, the overview presented in this Special Issue would not be complete without mentioning novel approaches for the characterization and modeling of nanomaterials for medical applications.

With this in mind, the goal of this Special Issue of the *International Journal of Molecular Sciences*, belonging to the “*Physical Chemistry and Chemical Physics*” Section and titled “*Nanomaterials in Cancer Diagnosis and Therapy*”, was to collect original research manuscripts that describe cutting-edge developments in nanomaterial technology for medicine and its translational applications, as well as reviews providing updates on the latest advancements in this field.

A total of **14** manuscripts have been submitted to the Special Issue, and **8** of them have been accepted for publication. The final collection includes **five** original research manuscripts and **three** reviews by authors from **five** different countries. A quick overview and general classification of the manuscripts are given below. 

## 2. Contributions

Various contributions to this Special Issue deal with research on “Cancer Diagnosis and Therapy”, innovative approaches for the treatment of oncological diseases, or more generally propose the optimization of the methods currently employed in several clinical practices. The attempt to reveal and understand the biological or radio–biological mechanisms governing such approaches is also often highlighted.

In the paper by Caizer et al. [7], the authors present a study based on computer simulations of superparamagnetic hyperthermia with CoFe_2_O_4_ ferrimagnetic nanoparticles coated with biocompatible gamma-cyclodextrins (γ-CDs) to be used in alternative cancer therapies with increased efficacy and nontoxicity. The specific loss power that leads to the heating of nanoparticles in superparamagnetic hyperthermia using CoFe_2_O_4_–γ-CDs was analyzed in detail, depending on the size of the nanoparticles, the thickness of the γ-CD layer on the nanoparticle surface, the amplitude and frequency of the alternating magnetic field, and the packing fraction of nanoparticles, in order to find the proper conditions in which the specific loss power is maximal. The authors found that the maximum specific loss power was determined by the Brown magnetic relaxation processes, and the maximum power obtained was significantly higher than what would be obtained by the Néel relaxation processes under the same conditions. The results reported in the paper show that nanoparticles of CoFe_2_O_4_-γ-CDs can be successfully used in superparamagnetic hyperthermia for alternative cancer therapies in order to increase their effectiveness on tumors and nontoxicity on healthy cells.

In the paper “*Radiolabeled Gold Nanoseeds Decorated with Substance P Peptides*: *Synthesis*, *Characterization and In Vitro Evaluation in Glioblastoma Cellular Models*” [8], by Silvia et al., the authors describe the synthesis and characterization of new nanoparticles (AuNP-SP and AuNP-SPTyr8) with a small gold core (~4 nm) that carry DOTA chelators obtained by the functionalization of AuNP-TDOTA with substance P (SP) peptides and SPTyr8 peptides, as well as studying their interaction with human serum albumin and human transferrin. The paper describes the radiolabeling of AuNP-SPTyr8 with ^125^I and ^177^Lu, as well as cellular uptake studies for the respective ^125^I-AuNP-SPTyr8 and ^177^Lu-AuNP-SPTyr8 in the U373 glioblastoma cell line. Finally, the authors report on microdosimetry simulations, which were performed with the aim of obtaining a better understanding of the experimental radiobiological results and assessing the possible contribution of the AuNPs’ gold core to radio-enhancement effects.

In their second paper, Professor Caizer et al. [9] propose an innovative strategy for the biocompatibility of these nanoparticles by using gamma-cyclodextrins (γ-CDs) to decorate the surface of magnetite (Fe_3_O_4_) nanoparticles. In this manuscript, the authors present and analyze in detail the influence of the biocompatible organic layer of cyclodextrins, from the surface of Fe_3_O_4_ ferrimagnetic nanoparticles, on the maximum specific loss power in superparamagnetic hyperthermia. This study allows for the practical implementation of superparamagnetic hyperthermia under optimal conditions using biocompatible Fe_3_O_4_ nanoparticles coated with γ-CDs dispersed in liquid in order to obtain maximum efficiency and a lack of cellular toxicity.

In “*Functionalization of Photosensitized Silica Nanoparticles for Advanced Photodynamic Therapy of Cancer*”, by Montero et al. [10], commercial and novel photosensitizers (PSs) based on BODIPY chromophores (haloBODIPYs and orthogonal dimers strategically designed with intense bands in the blue, green, or red region of the visible spectra as well as high singlet oxygen production) were covalently linked to mesoporous silica nanoparticles (MSNs) and then further functionalized with poly(ethylene glycol) (PEG) and folic acid (FA). Different combinations, varying the type of PS, the linkage design, and the length of the PEG, are detailed. The most promising PS-PEG-FA silica nanoplatforms, the biocompatibility in dark conditions, and the phototoxicity under various irradiation wavelengths at regulated light doses were compared with PSs free in solution and in vitro.

In the work by Nieto et al. [11], polydopamine nanoparticles (PDA-NPs) were loaded with Fe^3+^ at different pH values to assess the importance that the pH may have in determining their therapeutic activity and selectivity. In addition, doxorubicin was also loaded to the nanoparticles to achieve a synergist effect. The in vitro assays that were performed with two cell lines showed that, when Fe^3+^ was adsorbed in PDA-NPs at pH values close to which Fe(OH)_3_ begins to be formed, these nanoparticles had greater antitumor activity and selectivity despite having chelated a smaller amount of Fe^3+^. The results may constitute a good approach with which to target breast tumors.

Our Special Issue also covers some high-quality review articles. For instance, the review of Buddolla et al., “*Modernistic and Emerging Developments of Nanotechnology in Glioblastoma-Targeted Theranostic Applications*” [12], is based on the potential benefits of nanomaterials, which include their specificity, surface tunability, biodegradability, nontoxicity, and ligand functionalization; furthermore, near-infrared and photoacoustic imaging are sufficient in developing effective theranostics. This review discusses the recent developments in nanotechnology toward diagnoses, drug delivery, and therapy regarding glioblastoma.

The review of Jang et al., “*Nanoparticles Targeting Innate Immune Cells in Tumor Microenvironment*” [13], focuses on the recent studies on and challenges of using nanoparticles to target innate immune cells. In particular, the authors review the recent research lines where various NPs were used to control innate immune cells in the tumor microenvironment (TME), including tumor-associated macrophages (TAMs), dendritic cells (DCs), myeloid-derived suppressor cells (MDSCs), natural killer cells (NKs), and neutrophils. Understanding the cross-talk between tumors and innate immune cells could contribute to the development of strategies for manipulating the nanoparticles targeting tumor microenvironments.

Finally, in the review “*Electrochemical Sensors for Detection of Markers on Tumor Cells*” [14], electrochemical-cell-based biosensors used for tumor cell detection in the past five years were assessed. It can be argued that the use of new modifiers and new targets with which to improve the performance of sensors is a logical strategy for researchers. In addition, in recent years more studies have been carried out on combinations of a variety of monitoring technologies with which to prepare electrochemical cells based on biosensors, using optical and electrochemical technology, biochips, microfluidic technology, and DNA walker techniques. The combination of these new technologies provides advantages over traditional technologies. Unfortunately, existing electrochemical cell sensing technology has not been widely used in clinical practice. Current electrochemical cell sensing strategies lack the ability to detect intracellular protein markers. In addition, electrochemical-cell-based biosensors for the detection of living cells and single cells need to be developed; therefore, the application of electrochemical-cell-based biosensors in the detection of tumor cells needs to be further explored.

## 3. Conclusions

In conclusion, we were very pleased to guest edit this Special Issue, as it collects relevant contributions that reflect the increasingly widespread interest in nanomaterials and related applications in the field of cancer therapies and diagnostics. We hope that this Special Issue can reach the widest possible audience in the scientific community and contribute to further boosting scientific and technological advances in the intriguing world of nanomaterials as well as their multidisciplinary applications. Finally, we wish for this Special Issue to help its readers to conceive both new and improved ideas about “*Nanomaterials in Cancer Diagnosis and Therapy*” in their respective fields.
